# Sentinel node biopsy alone for breast cancer patients with residual nodal disease after neoadjuvant chemotherapy

**DOI:** 10.1038/s41598-021-88442-x

**Published:** 2021-04-27

**Authors:** Jung Whan Chun, Jisun Kim, Il Yong Chung, Beom Seok Ko, Hee Jeong Kim, Jong Won Lee, Byung Ho Son, Sei-Hyun Ahn, Sae Byul Lee

**Affiliations:** Division of Breast Surgery, Department of Surgery, University of Ulsan College of Medicine, Asan Medical Center, 88, Olympic-ro 43-gil, Songpa-Gu, Seoul, 05505 Republic of Korea

**Keywords:** Breast cancer, Cancer, Oncology

## Abstract

For residual N1 nodal disease following neoadjuvant chemotherapy (NAC) for patients with breast cancer, the optimal local therapy for axilla is an evolving area. We analyzed the long-term results of these patients according to axillary surgical methods using propensity score matching (PSM) to clarify whether omission of axillary lymph node dissection (ALND) is oncologically safe. This was a single institution retrospective study of patients with ypN1 from Asan Medical Center (AMC). We included 324 patients who had undergone axillary surgery with either sentinel lymph node biopsy (SLNB) only or ALND. The patients received NAC at AMC between 2008 and 2013. General indications for ALND included prominent nodes detected clinically before NAC, evident macrometastasis on multiple nodes during SLNB. Patients who had either micrometastasis or macrometastasis in 1 or 2 node(s) were included. SLNB was performed for patients with good responders to NAC with limited nodal burden. Patients were matched for baseline characteristics. After matching, we included 98 patients in each SLNB only group and ALND group respectively. We compared axillary recurrence-free survival (ARFS), distant metastasis-free survival (DMFS), overall survival (OS), and breast cancer-free survival (BCSS) according to the surgical method. The median follow-up period was 71 months. Univariate and multivariate analyses revealed no statistically significant differences between the two groups for ARFS, DMFS, OS, and BCSS. After the propensity score matching, no significant statistical differences were observed in 5-year ARFS, DMFS, OS, and BCSS between the SLNB only group and ALND group. SLNB might be a possible option for ALND in patients with breast cancer who have limited axillary node metastasis after NAC without compromising survival outcomes.

## Introduction

Over recent decades, the sentinel lymph node biopsy (SLNB) has been validated by several studies and has become the standard management for patients with clinically node-negative primary invasive breast cancer. Intraoperative frozen section analysis of sentinel lymph node has been shown to be accurate for the evaluation of axillary lymph node metastasis with high sensitivity and specificity^1^.

There has been a distinct trend for limiting the extent of axillary surgery to avoid the side effects from axillary lymph node dissection (ALND), which is known to be associated with a higher morbidity, prolonged hospitalization, and impairment of quality of life. Some studies have also been focused on the safe omission of complete ALND when the axillary nodes contain micro or macro-metastasis. The result of the ACOSOGZ0011 trial showed that ALND was not needed in women with early breast cancer with only one or two sentinel node metastases who would receive whole breast radiation as part of breast-conserving surgery^2^. A recent randomized controlled trial confirmed the oncologic safety of omitting complete ALND for micro-metastasis-positive SLNB (IBCSG 23-01**)**. The results of this trial show that in patients with only micrometastases in the sentinel nodes, ALND is not needed. Both aforementioned studies showed that patients with limited sentinel lymph node metastasis who were treated with BCS, whole breast radiation therapy, and adjuvant systemic treatment, could be spared an ALND without compromising locoregional control or survival.

Additionally, omission of ALND in patients with cN1 disease who are found to be ypN0 after neoadjuvant chemotherapy has shown acceptable results throughout several trials (ACOSOGZ1071, SENTINA, and SN-FNAC). The publication of these trials has resulted in changes in practice for a majority of surgeons according to a survey^3^. The SLNB after neoadjuvant therapy for patients who are ypN0 has shown similar results in a retrospective study with respect to survival or recurrence^4^.

However, the appropriateness of substituting SLNB for ALND in patients with ypN1 breast cancer following neoadjuvant chemotherapy is yet to be determined. We compared the oncologic outcome following SNB only or ALND in patients with breast cancer who had 1 or 2 residual nodal metastases after neoadjuvant chemotherapy.

## Methods

### Patients

We reviewed the records of patients who had undergone neoadjuvant chemotherapy followed by surgery at Asan Medical Center (AMC) from 2008 and 2013. Of the 1957 patients, we excluded patients' record if they had bilateral breast cancer, concurrent other types of cancer during the follow-up period, distant metastasis at the time of diagnosis. We further narrowed down to patients who were revealed to have 1 or 2 positive axillary lymph nodes after surgery.

Total of 324 eligible patients were included for further analysis. Patients were categorized to SLNB alone group or ALND group regardless of the breast surgery type. Clinical and pathologic data, which include age at diagnosis, tumor histologic or nuclear grade, hormone receptor, HER2 status, initial clinical stage, pathologic stage, number of positive lymph nodes for malignancy, surgical methods, types of adjuvant treatment modalities, type of recurrence, and follow-up period were obtained. The clinical and pathologic stage classification was based on the definitions presented in the 8^th^ edition of the American Joint Committee on Cancer staging system. Due to the retrospective study design, the informed consent was waived by and was approved by the Institutional Review Board of Asan Medical Center, Seoul, South Korea (20,171,341) and performed in accordance with the principles of the Declaration of Helsinki. AMC multidisciplinary breast cancer team has established and actively participated in the decision-making process for patients. The team consists of breast surgeons, oncologists, and radiation oncologists that has had a regular meeting on a weekly basis.

### Preoperative chemotherapy

Neoadjuvant chemotherapy was delivered every 3 weeks, the standard regimens were chosen based on the clinical stage of patients. Although the regimens evolve continuously, the oncologists of our institution generally followed the most recent NCCN guideline of the time treatment delivered. Surgery was performed at 3 to 4 weeks after final scheduled chemotherapy. For HER2-positive patients, trastuzumab-based chemotherapy was provided before surgery and completed trastuzumab therapy without chemotherapy after surgery, with total of 18 administrations of trastuzumab from the beginning and the end of scheduled course. Responses to NAC of our patients were confirmed using either ultrasonogram or MRI of breast.

### Surgical procedures

We performed either total mastectomy (n = 160) or breast conserving surgery (n = 164) to our patients. We followed the standard total mastectomy procedure with preservation of pectoralis minor and major muscles. The breast conserving surgery was performed according to general standards with securing safety margin. We used 99 m Tc-sulfur colloid for radiopharmaceutical agent with gamma probe detection for SLN identification. Also clinically enlarged, palpable axillary lymph nodes without intense radioactive signal were also excised along with SLN and included with total number of SLNB. As a general rule, range of axillary dissection were level II when we defined the ALND in our study. Also, we generally performed to ALND for patients with positive SLNB. Although each of 8 surgeons’ threshold for proceeding to ALND or not was not always uniform, SLNB was performed for patients with good responders to NAC with limited nodal burden based on surgeon’s decision. We included 98 patients in each SLNB only group and ALND group respectively after matching. ypN1 patients had either micrometastasis or macrometastasis and we chose patients with only 1 or 2 positive node(s) on final pathology report.

### Adjuvant therapy

Standard adjuvant therapy was performed to all patients according to their clinical status. Adjuvant endocrine therapy was provided for hormone receptor positive patients. All patients who were undergone breast conserving surgery received whole-breast and axilla radiotherapy (RT) with conventional fractionation. The use of adjuvant radiotherapy (RT) and RT fields was determined based on patient and tumor characteristics and the physician’s preferences. All patients who were undergone breast conserving surgery received adjuvant RT to the ipsilateral breast. The clinical high-risk was defined as two or more of the following risk factors: young age (≤ 40), histologic grade 3, hormone receptor negative, high proliferative index (Ki-67 ≥ 14%), and presence of LVI. If patients had clinical high-risk features, they received regional nodal irradiation from axillary level 1 up to supraclavicular area regardless of the extent of axillary dissection in both groups. Patients who had N1 and clinical high-risk received RT targeting the ipsilateral breast or chest wall, and regional nodal irradiation including the axillary apex and supraclavicular fossa with or without the internal mammary chain.

### Statistical analysis

The primary endpoint was the time of the disease recurrence as the first event. Axillary recurrence-free survival (ARFS) was defined as the time from the date of the initial surgery to the date of the confirmation of an initial ipsilateral axillary recurrence. The distant metastasis-free survival (DMFS) was defined as the interval from operation to distant metastasis, overall survival (OS) was defined as the interval from the initial surgery to the time of death, and breast cancer-specific survival (BCSS) was defined as the interval from the initial surgery to the time of breast cancer-related death specifically.

The independent *t*-test was utilized to compare the continuous variables, and the chi-square test and Fisher’s exact test were applied to compare clinicopathologic factors. The Kaplan–Meier method was used to generate survival plots, and the statistical significance of survival differences among selected factors was verified with the log-rank test. The cox proportional hazards model was applied to univariate and multivariate analysis, to estimate the hazard ratios (HRs) and 95% confidence intervals (CIs). In an attempt to minimize the potential bias between the subgroups, we applied propensity score matching (PSM) for further data analysis. The covariates included in the process were age at initial operation, clinical T stage, N stage, number of positive node, surgical method, tumor grade, lympho-vascular invasion status, hormone receptor status, HER2 status, Ki-67 level, radiation therapy, and endocrine therapy. The multivariate logistic regression model was used to calculate the propensity scores for each of the patients. A two-sided p value of less than 0.05 was considered statistically significant. All statistical analyses were performed with SPSS statistics version 23.0 (IBM Corp., Armonk, USA).

## Results

### Patients’ baseline characteristics

The patients’ characteristics are presented in Table 1. The age at initial operation (n = 324) was 45.5 ± 9.3 years. We recruited patients with only 1 or 2 pathologically positive nodes in which 202 (62.3%) of them had only one metastatic node. For the hormone receptor status, estrogen receptor was positive in 224 (69.1%) patients, and progesterone receptor was positive in 127 (39.2%) patients. HER2 positivity was found in 88 (27.2%) patients. For the adjuvant management, 222 (68.5%) patients underwent the adjuvant endocrine therapy, while 230 (71.0%) patients received radiation therapy. The mean number of excised sentinel lymph nodes was 4.20 in the SLNB only group and 4.26 in the ALND group (*p* = 0.767). Three to five sentinel lymph nodes were harvested for a majority of the patients (84% in the SLNB only group and 69.5% in the ALND group). Among the removed lymph nodes, the largest invasion depth was 4.7 mm in the SLNB only group and 7.63 mm in the ALND group (*p* = 0.114) (Table 2).

**Table 1:** Baseline patient characteristics before matching.

	Total	SLNB only	ALND	*P*-value
		N = 106	N = 218	
		N (%)	N (%)	
**Age at diagnosis (years)**				0.126
50 ≥	220 (67.9)	78 (73.6)	142 (65.1)	
50 <	104 (32.1)	28 (26.4)	76 (34.9)	
**Initial clinical T stage**				0.156
1	19 (5.9)	8 (7.5)	11 (5.0)	
2	236 (72.8)	82 (77.4)	154 (70.6)	
3	58 (17.9)	15 (14.2)	43 (19.7)	
4	11 (3.4)	1 (0.9)	10 (4.6)	
**Initial clinical N stage**				0.682
0	81 (25.0)	28 (26.4)	53 (24.3)	
1	243 (75.0)	78 (73.6)	165 (75.7)	
**Pathologic tumor size (cm, SD)**	2.29 (1.73)	1.89 (1.27)	2.50 (1.88)^`^	0.001
**Number of metastatic node(s)**				< 0.001
1	202 (62.3)	82 (77.4)	120 (55.0)	
2	122 (37.7)	24 (22.6)	98 (45.0)	
**Breast surgery**				0.082
Breast conserving surgery	164 (50.6)	61 (57.5)	103 (47.2)	
Mastectomy	160 (49.4)	45 (42.5)	115 (52.8)	
**Histologic grade**				0.813
1	13 (4.0)	3 (2.8)	10 (4.6)	
2	212 (65.4)	68 (64.2)	144 (66.1)	
3	94 (29.0)	33 (31.1)	61 (28.0)	
Unknown	5 (1.5)	2 (1.9)	3 (1.4)	
**Nuclear grade**				0.786
1	11 (3.4)	2 (1.9)	9 (4.1)	
2	213 (65.7)	71 (67)	142 (65.1)	
3	95 (29.3)	32 (30.2)	63 (28.9)	
Unknown	5 (1.5)	1 (0.9)	4 (1.9)	
**LVI**				0.13
Absent	193 (59.6)	66 (62.3)	127 (58.3)	
Present	123 (38.0)	40 (37.7)	83 (38.1)	
Unknown	8 (2.5)	0 (0)	8 (3.7)	
**ER**				0.143
Negative	100 (30.9)	27 (25.5)	73 (33.5)	
Positive	224 (69.1)	79 (74.5)	145 (66.5)	
**PR**				0.186
Negative	197 (60.8)	59 (55.7)	138 (63.3)	
Positive	127 (39.2)	47 (44.3)	80 (36.7)	
**HER2 status**				0.313
Negative	236 (72.8)	81 (76.4)	155 (71.1)	
Positive	88 (27.2)	25 (23.6)	63 (28.9)	
**Ki67**				0.133
20 ≥	239 (73.8)	81 (76.4)	158 (72.5)	
20 <	77 (23.8)	25 (23.6)	52 (23.9)	
Unknown	8 (2.5)	0 (0)	8 (3.7)	
**Radiation therapy**				0.558
No	94 (29.0)	33 (31.1)	61 (28.0)	
Yes	230 (71.0)	73 (68.9)	157 (72)	
**Endocrine therapy**				0.171
No	102 (31.5)	28 (26.4)	74 (33.9)	
Yes	222 (68.5)	78 (73.6)	144 (66.1)	

SLNB, sentinel lymph node biopsy; ALND, axillary lymph node dissection; SD, standard deviation; LVI, lymphovascular invasion; ER, estrogen receptor; PR, progesterone receptor; HER2, human epidermal growth factor receptor type 2.

**Table 2:** Surgically removed axillary lymph node status.

	SLNB only	ALND	*P*-value
	N (%)	N (%)	
**Number of sentinel nodes**			0.053
1	1 (0.9)	3 (1.9)	
2	7 (6.6)	14 (9.1)	
3	20 (18.9)	40 (26.0)	
4	29 (27.4)	27 (17.5)	
5	40 (37.7)	40 (26.0)	
> 5	9 (8.4)	30 (18.9)	
Mean (standard deviation)	4.2 (1.12)	4.26 (1.55)	0.767
**Largest invasion depth (mm)**	4.7	7.63	0.114

SLNB, sentinel lymph node biopsy; ALND, axillary lymph node dissection.

### Comparison of characteristics according to surgical methods

There were 324 eligible patients: 106 in the SLNB only group and 218 in the ALND group. In order to minimize other confounding variables, the patients were further stratified to match baseline characteristics of Table 1. Finally, we analyzed the data of 98 patients in both matched groups. All the covariates were balanced after matching (Table 3).

**Table 3:** Comparison of characteristics of patients who underwent SLNB and ALND after propensity score matching.

	SLNB only	ALND
	N = 98	N = 98
	N (%)	N (%)
**Age at diagnosis (years)**		
50 ≥	71 (72.4)	67 (68.3)
50 <	27 (27.5)	31 (31.6)
**Initial clinical T stage**		
1	8 (8.1)	7 (7.1)
2	74 (75.5)	76 (77.5)
3	15 (15.3)	13 (13.2)
4	1 (1.0)	2 (2.0)
**Initial clinical N stage**		
0	23 (23.4)	26 (26.5)
1	75 (76.5)	72 (73.4)
**Pathological node**		
1	74 (75.5)	75 (76.5)
2	24 (24.4)	23 (23.4)
**Breast surgery**		
Breast conserving surgery	56 (57.1)	57 (58.1)
Mastectomy	42 (42.8)	41 (41.8)
**Histologic grade**		
1	2 (2.0)	5 (5.1)
2	66 (67.3)	63 (64.2)
3	29 (29.5)	29 (29.5)
Unknown	1 (1.0)	1 (1.0)
**Nuclear grade**		
1	2 (2.0)	4 (4.0)
2	66 (67.3)	64 (65.3)
3	29 (29.5)	29 (29.5)
Unknown	1 (1.0)	1 (1.0)
**LVI**		
Absent	62 (63.2)	65 (66.3)
Present	36 (46.7)	32 (32.6)
Unknown	0 (0.0)	1 (1.0)
**ER**		
Negative	24 (24.4)	24 (24.4)
Positive	74 (75.5)	74 (75.5)
**PR**		
Negative	55 (56.1)	54 (55.1)
Positive	43 (43.8)	44 (44.9)
**HER2 status**		
Negative	74 (75.5)	78 (79.5)
Positive	24 (24.4)	20 (20.4)
**Ki67**		
20 ≥	76 (77.5)	75 (76.5)
20 <	22 (22.4)	23 (23.4)
**Radiation therapy**		
No	30 (30.6)	32 (32.6)
Yes	68 (69.3)	66 (67.3)
**Endocrine therapy**		
No	25 (25.5)	26 (26.5)
Yes	73 (74.4)	72 (73.4)

SLNB, sentinel lymph node biopsy; ALND, axillary lymph node dissection; LVI, lymphovascular invasion; ER, estrogen receptor; PR, progesterone receptor; HER2, human epidermal growth factor receptor type 2.

### Survival outcomes

**Figure 1:**
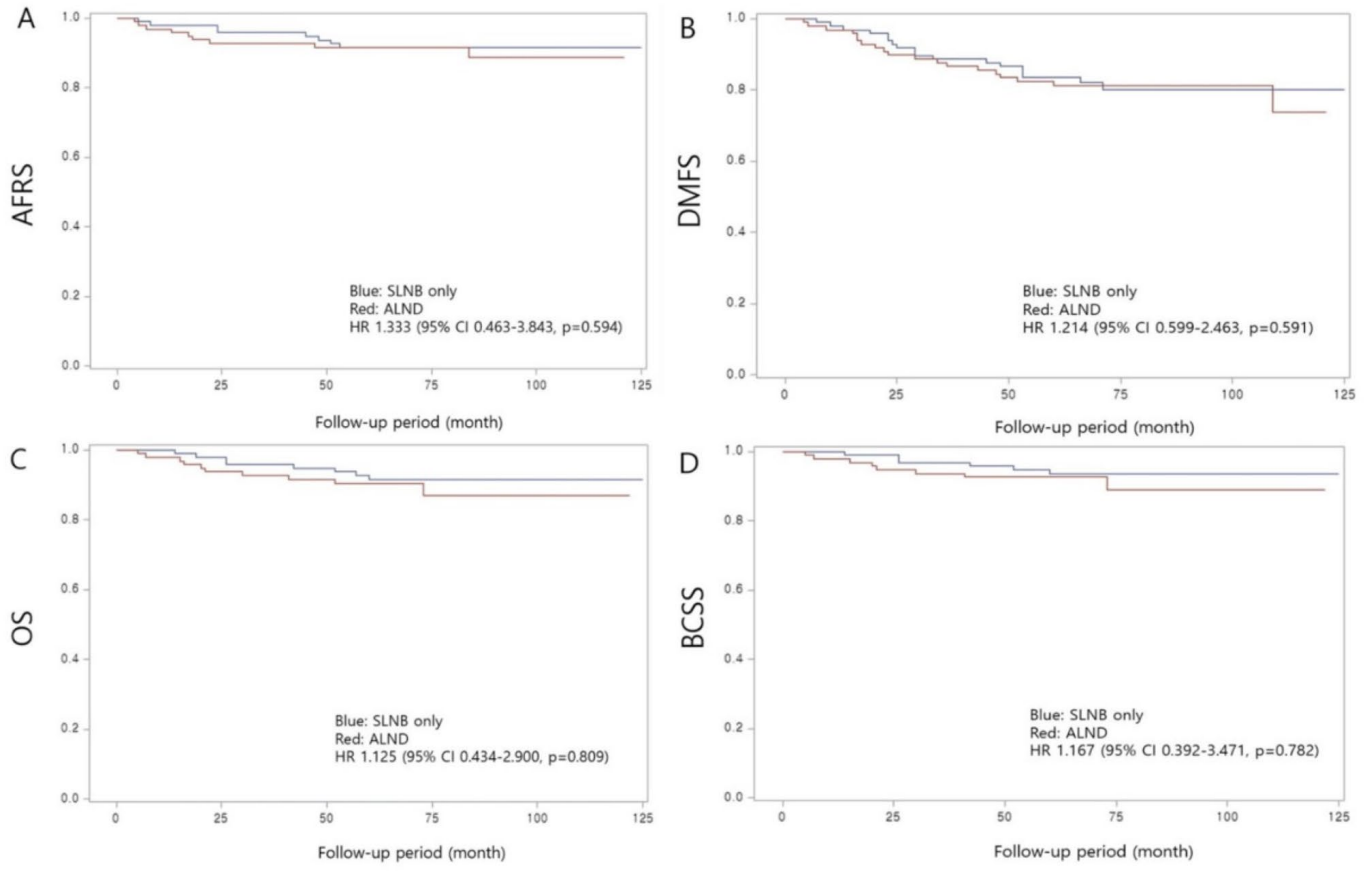
Before matching, Unadjusted Kaplan–Meier survival analysis of (**A**) axillary recurrence-free survival (ARFS), (**B**) distant metastasis-free survival (DMFS), (**C**) overall survival (OS), and (**D**) breast cancer-specific survival (BCSS) between the sentinel lymph node biopsy (SLNB) only and axillary lymph node dissection (ALND) groups.

The median follow-up period was 71 months (range: 5.0–142 months). Before matching, Kaplan–Meier analysis revealed no significant differences between the SLNB alone group and the ALND group with respect to 5-year ARFS (91.2% vs. 91.4%,* p* = 0.594), DMFS (84.6% vs.83.2%,* p* = 0.591), OS (92.1% vs. 91.1%, *p* = 0.809), and BCSS (94.0% vs. 92.0%,* p* = 0.782) (Fig. 1).

The left 2 columns of the Table 4 show the results of the univariate and multivariate analyses prior to matching. In both the univariate and multivariate analysis, no significant differences were observed in ARFS, DMFS, OS, and BCSS between the two groups.

**Table 4:** Cox regression analysis of ARFS, DMFS, OS, and BCSS.

Variables	No. of events	Univariate		Multivariate^a^		PSM^b^	
		HR (95% CI)	*p* value	HR (95% CI)	*p* value	HR (95% CI)	*P* value
**ARFS**							
SLNB only	9	1 (Ref)		1 (Ref)		1 (Ref)	
ALND	19	1.043 (0.471–1.308)	0.918	1.021 (0.422–2.470)	0.963	1.333 (0.463–3.843)	0.594
**DMFS**							
SLNB only	18	1 (Ref)		1 (Ref)		1 (Ref)	
ALND	41	1.105 (0.634–1.925)	0.725	0.949 (0.515–1.749)	0.867	1.214 (0.599–2.463)	0.591
**OS**							
SLNB only 8	1 (Ref)		1 (Ref)		1 (Ref)		
ALND	21	1.301 (0.576–2.939)	0.527	0.967 (0.383–2.440)	0.943	1.125 (0.434–2.900)	0.809
**BCSS**							
SLNB only	6	1 (Ref)		1 (Ref)		1 (Ref)	
ALND	20	1.605 (0.643–4.003)	0.311	1.318 (0.453–3.835)	0.613	1.167 (0.392–3.471)	0.782

^a^Adjusted for variables listed in the Table 1.

^b^Column PSM is the result of a univariate analysis after propensity matching.

ARFS, axillary recurrence-free survival; DMFS, distant metastasis-free survival; OS, overall survival; BCSS, breast cancer-specific survival; SLNB, sentinel lymph node biopsy; ALND, axillary lymph node dissection.

**Figure 2:**
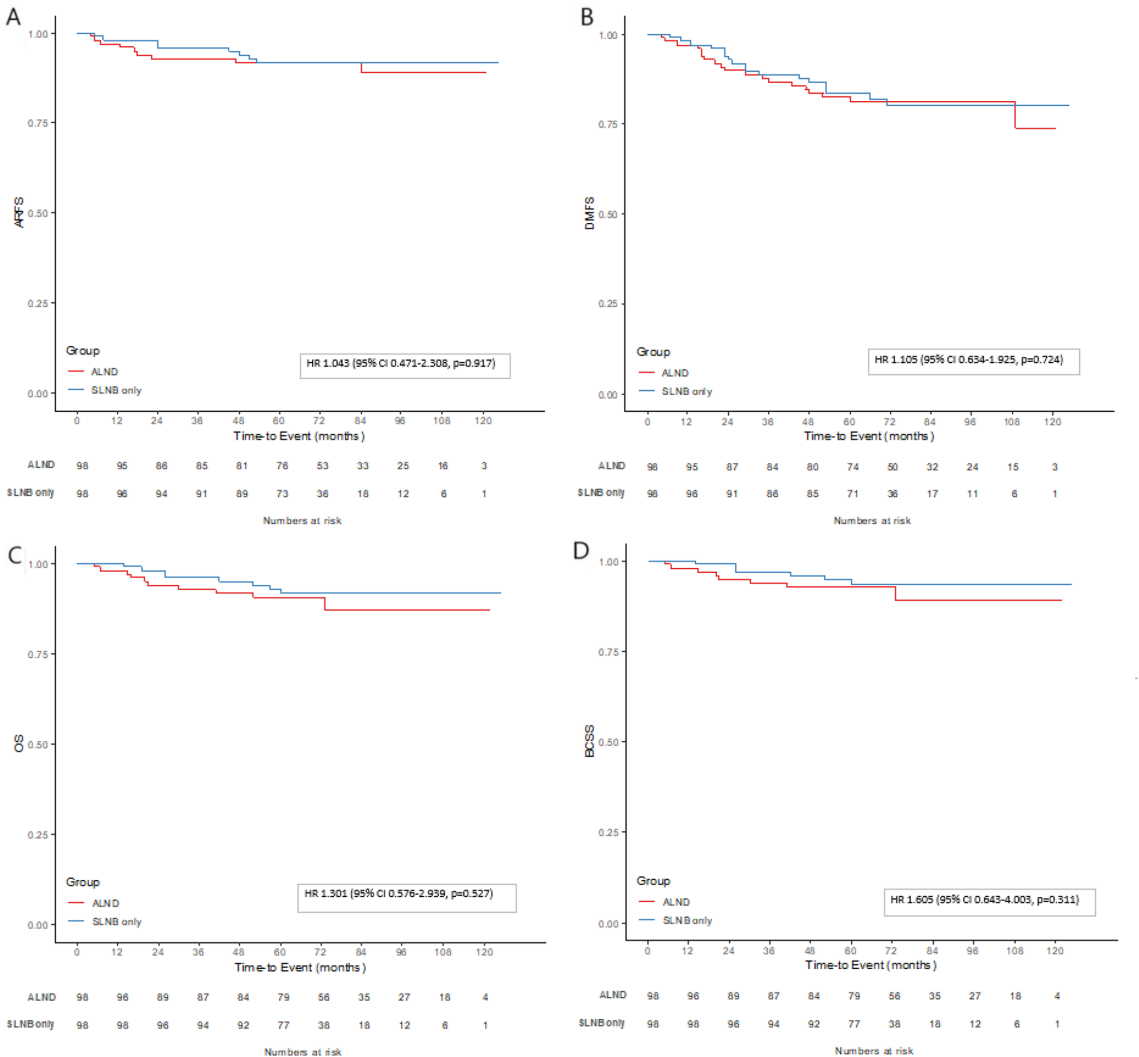
Propensity score-matched Kaplan–Meier survival analysis of (**A**) axillary recurrence-free survival (ARFS), (**B**) distant metastasis-free survival (DMFS), (**C**) overall survival (OS), and (**D**) breast cancer-specific survival (BCSS) between the sentinel lymph node biopsy (SLNB) only and axillary lymph node dissection (ALND) groups.

After matching, the Kaplan–Meier analysis revealed no significant differences between the SLNB alone group and the ALND group with respect to 5-year ARFS (91.6% vs. 91.7%, *p* = 0.917), DMFS (83.5% vs. 81.3%,* p* = 0.724), OS (93.6% vs. 92.7%, *p* = 0.527), and BCSS (93.6% vs. 92.7%,* p* = 0.311) (Fig. 2).

In order to further clarify the factors that may influence the outcome, we performed a multivariate Cox regression analysis of matching data (Table 5). For ARFS, pathologic lymph node metastasis were negatively related to the outcome. Also, increased risk of recurrence was observed when radiation therapy was omitted. The factors that showed significant correlation with DMFS were the pathologic tumor size, Ki-67 status, and radiation therapy. The OS was significantly correlated with cT stage, HER2 status, and Ki-67 status. Furthermore, cT stage, pathologic tumor size, HER2 status, Ki-67 status, and radiation therapy were significant factors for BCSS. In addition, increased risk of recurrence was observed when radiation therapy was omitted in regards of ARFS, DMFS and BCSS. The ARFS, DMFS, OS, and BCSS did not show significant differences between either of the axillary surgery methods. The data of the molecular subtypes and OS analysis between SLNB an ALND group was provided in the supplementary information.

**Table 5:** Multivariate Cox regression analysis after propensity score matching.

	ARFS		DMFS		OS		BCSS	
Variables	HR (95% CI)^a^	*p* value	HR (95% CI) ^a^	*p* value	HR (95% CI) ^a^	*p* value	HR (95% CI) ^a^	*p* value
cT stage (cT1, 2 vs. cT3, 4)	1.79 (0.46–7.11)	0.402	2.26 (0.88–5.79)	0.089	4.69 (1.40–15.7)	**0.012**	19.0 (3.79–95.3)	**0.000**
cN stage (cN0 vs. cN1)	0.70 (0.21–2.34)	0.558	1.14 (0.51–2.57)	0.756	1.84 (0.53–6.40)	0.338	1.79 (0.45–7.23)	0.412
Breast surgery (BCS vs. TM)	0.31 (0.04–2.36)	0.255	0.35 (0.09–1.45)	0.149	0.89 (0.12–6.64)	0.91	0.16 (0.01–2.55)	0.195
Axilla surgery (SLNB vs. ALND)	1.20 (0.41–3.54)	0.741	0.85 (0.42–1.72)	0.645	1.07 (0.39–2.96)	0.894	1.39 (0.40–4.81)	0.605
Pathologic tumor size	1.46 (0.96–2.20)	0.074	1.41 (1.07–1.85)	**0.015**	1.34 (0.91–1.97)	0.136	1.53 (0.91–2.57)	**0.011**
Pathologic node metastasis	5.89 (1.95–17.8)	**0.002**	1.17 (0.53–2.56)	0.07	1.99 (0.72–5.48)	0.183	2.48 (0.71–8.69)	0.157
Histologic grade (1, 2 vs. 3)	2.63 (0.00–3236)	0.79	5.19 (0.63–42.7)	0.126	0.53 (0.00–36,003)	0.91	0.245 (0.00–40,375)	0.915
Nuclear grade (1, 2 vs. 3)	0.75 (0.00–930)	0.936	0.16 (0.01–1.09)	0.06	0.97 (0.00–67,452)	0.997	3.09 (0.00–5.321E)	0.932
Lymphatic invasion (neg. vs. pos.)	0.73 (0.21–2.55)	0.621	0.88 (0.42–1.84)	0.734	1.01 (0.36–2.87)	0.973	1.13 (0.33–3.84)	0.843
Estrogen receptor (neg. vs. pos.)	0.00 (0.00–9.85E)	0.931	0.00 (0.00–11E)	0.913	0.00 (0.00–1.755E)	0.899	0.00 (0.00–1.649E)	0.919
Progesterone receptor (neg. vs. pos.)	0.83 (0.20–3.36)	0.788	1.63 (0.57–4.68)	0.367	1.23 (0.21–7.27)	0.823	2.74 (0.18–42.8)	0.473
HER2 status (neg. vs. pos.)	0.76 (0.17–3.32)	0.716	0.39 (0.14–1.09)	0.072	0.14 (0.03–0.74)	**0.02**	0.02 (0.00–0.30)	**0.005**
Ki-67 (20 ≥ vs. 20 <)	3.06 (0.66–14.2)	0.154	4.13 (1.55–11.0)	**0.005**	4.26 (1.03–17.58)	**0.045**	6.91 (1.05–45.3)	**0.044**
Radiation therapy (No vs. Yes)	0.10 (0.01–0.67)	**0.018**	0.26 (0.07–0.98)	**0.046**	0.23 (0.04–1.44)	0.117	0.05 (0.00–0.77)	**0.032**
Endocrine therapy (No vs. Yes)	1423 (0.00–4.47E)	0.94	3485 (0.00–6.92E)	0.93	793.9 (0.00–1.718E)	0.928	400 (0.00–1.339E)	0.951

ARFS, axillary recurrence-free survival; DMFS, distant metastasis-free survival; OS, overall survival; BCSS, breast cancer-specific survival; BCS, breast conserving surgery; TM, total mastectomy; SLNB, sentinel lymph node biopsy; ALND, axillary lymph node dissection.

Bold values indicate statistically significant *p*-value less than 0.05.

## Discussion

Traditionally ALND has been the standard surgical choice for patients with primary breast cancer or node-positive disease after NAC. Because ALND is associated with a significant risk of well-known complications such as lymphedema and peripheral neurologic sequelae, there has been a trend toward less extensive axillary surgery . SLNB has replaced axillary evaluations for patients with primary breast cancer who have clinically negative nodes^5^. Several recent trials have provided evidence for further limiting the indication of ALND. The results of the ACOSOG Z0011 and the IBCSG 23–01 trials show that ALND is not needed in women with early stage breast cancer with limited axillary nodal burden^2,6^.

However, more evidence is needed to validate a reduction in the extent of axillary surgery for SLNB in patients who are clinically node-positive and have been treated with neoadjuvant chemotherapy. An important issue regarding the use of SLNB only instead of ALND is the reliability of the procedure and its results. Initial concerns about the feasibility of sentinel lymph node biopsy following neoadjuvant chemotherapy have been based on potentially altered lymphatic drainage after chemotherapy and possible nonuniform response of tumor burden from the treatment^7^. The false-negative rate has been a major issue, especially for patients with clinically node-positive disease. The ACOSOG Z1071 clinical trial showed that the false-negative rate of SLNB after NAC in patients with cN1 and at least 2 sentinel nodes identified during surgery was 12.6%^8^. Among the 343 patients who had undergone both SLNB and ALND, the overall false-negative rate was 10.7%, with no significant difference according to pretreatment node status (*p* = 0.51)^9^. Another study on the reliability of SLNB following neoadjuvant chemotherapy revealed a false-negative rate of 13% and a sentinel lymph node identification rate of 91%^10^. However, according to the SENTINA study, the false-negative rate was 7% or less when three or more sentinel nodes were removed, compared to 19% with two nodes removed and 24% when only one node removed^11^. In the present study, the mean number of the harvested sentinel lymph nodes was 4.20 for the SLNB only group and 4.26 for the ALND group. Also, because the majority of both groups had three or more axillary nodes removed (92.5% vs 90.0%), SLNB could be considered reliable based on the prior research results.

Several trials have been trying to prove the feasibility of omitting ALND for patients with limited axillary tumor burden, especially for ypN0 cases following neoadjuvant chemotherapy. There has been increasing acceptance of omitting ALND following neoadjuvant chemotherapy in patients with cN1 disease who have been found to have a negative SLNB. According to Caudle et al., 55.9% of surgeons who are familiar with the ACOSOGZ1071, SENTINA, and SN-FNAC trials are currently offering SLNB to > 50% of their patients with planned omission of ALND if SLNB is negative even though they are initially node-positive^3^.Kang et al. reviewed 1,247 patients who had breast cancer with clinical conversion of axillary nodes from positive to negative after neoadjuvant chemotherapy. Of these patients, 819 patients in the ALND group and 428 patients in the SLNB group had similar axillary and distant recurrence-free survival^12^.

Patients treated with neoadjuvant chemotherapy who are at least ypN1 at SLNB are considered to have residual nodal disease, and ALND still remains the standard of care. To the best of our knowledge, few studies have addressed the safety of SLNB only for patients with ypN1 breast cancer, especially with 1 or 2 positive lymph nodes. In the present study, the omission of ALND was not associated with an inferior outcome in both non-matched and matched analyses in terms of ARFS, DMFS, OS, and BCSS. The results showed a lack of significant differences in outcomes between the SLNB only group and the ALND group in residual nodal disease after neoadjuvant chemotherapy. A recent retrospective study of 1,617 ypN1 patients (ALND, 1,313; SLND, 304) after neoadjuvant chemotherapy reached a rather conservative conclusion^13^. According to their data, the SLNB group showed significantly low survival in both univariate and multivariate analyses (HR = 1.7; 95% CI = 1.3–2.2, *p* < 0.001), with an estimated 5-year OS of 71%, compared with 77% in the ALND group (*p* = 0.01). However, there are differences in subsets of patients. Over 20% of patients had three positive lymph nodes, and this group of patients were excluded in our study. Furthermore, nearly 30% of patients were in stage 3 compared with our study population of 15% after the propensity score matching. In addition, the results of their specific subset of patients were similar to those of our study. They performed a matched subgroup analysis for patients with luminal A or B tumors and residual disease in a single lymph node. The results from the SLNB only and ALND groups were equivalent in this subset of patients (HR = 1.03, 95% CI = 0.59–1.8, *p* = 0.91). Also, a Kaplan–Meier analysis showed similar survival rates between the two groups, with an estimated 5-year OS of 85% and 82% for SLNB only and ALND, respectively (*p* = 0.88). In our study, the survivals were no different either before or after matching patients. We think that we recruited eligible patients who had only 1 or 2 metastatic nodes for analysis before matching. Among a number of variables we matched for patients’ details, the number of affected nodes may be one of the most influential factor for the survivals. Also, adjuvant therapy might be effective enough to override the baseline differences for patients with residual 1–2 nodal disease after NAC.

The multivariate analysis after matching of this study, performed to clarify the factors that exhibit significant influence on the oncologic outcome, revealed that radiation therapy was involved with improved ARFS, DMFS, and BCSS. Among other factors, the pathologic node metastasis was significant only for ARFS. We recruited patients’ data with low burden axillary disease, with only 1–2 lymph node metastasis. Thus, pathologic node, the well-known prognostic factor might not be revealed to be significant for DMFS, OS and BCSS. Our data also suggested that radiation therapy is significantly related with survival, while the univariate, multivariate, and propensity score-matched data exhibited no statistical differences between the SLNB only and ALND groups. As shown in Table 5, radiotherapy treatment was correlated with a low risk of ARFS (HR = 0.10, *p* = 0.018), DMFS (HR = 0.26, *p* = 0.046), and BCSS (HR = 0.05, *p* = 0.032) after matching. According to Donker et al., axillary radiotherapy can be considered as an effective alternative to ALND with acceptable outcome^14^. While the role of post-mastectomy radiation therapy in cN1 patients is still an area of investigation, one retrospective analysis of 15,315 cases (mastectomy, 10,283; breast-conserving surgery, 5,032; both ypN0 or ypN +) from the national cancer database has addressed this issue. In a subset analysis, OS was improved with post-mastectomy radiation therapy in the ypN1 subgroup (*p* < 0.05^15^. Among ongoing trials, a randomized phase III trial has been comparing ALND to axillary radiation in patients with breast cancer (cT1-3 N1) who have positive SLN after receiving neoadjuvant chemotherapy (AlllianceA011202). One arm comprises of ALND with nodal radiation (ALN level III and supraclavicular fossa) but no radiation to dissected axilla (ALN levels I–II), while the other arm is axillary radiation plus nodal irradiation (ALN levels I–III and supraclavicular fossa). These results should help clarify the role of axillary irradiation and thus the appropriate surgical choice for patients with ypN1 breast cancer.

Our study has several limitations. First, this study was retrospective in nature and was conducted at a single institution. And we admit that it is possible for the data to involve potential selection bias for eligible SLNB only group who might be good responders to preoperative chemotherapy. Because the patients’ clinical response to chemotherapy may clearly influence a surgeon’s decision whether to directly proceed to ALND or not. Hence, in order to control other confounding variables, we conducted a propensity score matching to reduce the selection bias. Also, these data were collected from 8 breast surgeons of AMC and we share same protocol about surgical approach based on the NCCN guideline, but not all surgeons had the exact same threshold to make decisions whether to perform ALND or not. Furthermore, we generally include clinically enlarged but without gamma signal, non-sentinel lymph nodes to the SLNB procedure so that the number of excised nodes for SLNB group may be relatively high. On the other hand, the fact that the data was collected from surgeons of diverse opinion on the axillary surgery may be a partial reflection on the actual clinical practice. Because, in the real clinical practice, each surgeon has his or her own perspective and threshold for axillary surgery. Finally, in the absence of randomized controlled trials about this issue, we believe our study might be an addition of supporting evidence for reducing axillary surgery extent while minimizing ALND-associated morbidity and preserving patients’ quality of life.

In conclusion, omission of ALND for 1 or 2 positive sentinel lymph nodes after neoadjuvant chemotherapy may not compromise locoregional control or survival. The results of this study demonstrated that the ALND-associated morbidity might be avoided without decreasing cancer control in patients with limited nodal burden after neoadjuvant chemotherapy. However, final treatment decisions should always be made in the context of a multidisciplinary setting to maximize regional control and minimize treatment related morbidity (Supplementary Information).

## Supplementary Information

Supplementary Information
