# Supplementary material for: Sentinel node biopsy alone for breast cancer patients with residual nodal disease after neoadjuvant chemotherapy
Source: Sci Rep. 2021 Apr 27;11:9056. doi: 10.1038/s41598-021-88442-x (PMC8079673; doi:10.1038/s41598-021-88442-x)

**Sentinel node biopsy alone for breast cancer patients with residual nodal disease after neoadjuvant chemotherapy**

Jung Whan Chun, Jisun Kim, Il Yong Chung, Beom Seok Ko, Hee Jeong Kim, Jong Won Lee, Byung Ho Son, Sei-Hyun Ahn, and Sae Byul Lee^*^

Division of Breast Surgery, Department of Surgery, University of Ulsan College of Medicine, Asan Medical Center, Seoul, Republic of Korea

^*^Corresponding author:

Sae Byul Lee

Division of Breast Surgery, Department of Surgery, University of Ulsan College of Medicine, Asan Medical Center, 88, Olympic-ro 43-gil, Songpa-Gu, Seoul, 05505, Republic of Korea. Tel: +82-2-3010-1729; Fax: +82-2-474-9027; E-mail: newstar153@hanmail.net

ORCID ID: https://orcid.org/0000-0002-3370-6937

Supplementary material :

Molecular subtypes and Overall Survival (OS) analysis between SLNB only and ALND group.

| subtype | | Total N | N of Events | Censored | |
| --- | --- | --- | --- | --- | --- |
|  |  |  |  | N | Percent |
| LumA | SLNB only | 65 | 1 | 64 | 98.5% |
|  | ALND | 128 | 7 | 121 | 94.5% |
|  | Overall | 193 | 8 | 185 | 95.9% |
| LumB | SLNB only | 19 | 1 | 18 | 94.7% |
|  | ALND | 24 | 0 | 24 | 100.0% |
|  | Overall | 43 | 1 | 42 | 97.7% |
| HER2 | SLNB only | 9 | 2 | 7 | 77.8% |
|  | ALND | 31 | 5 | 26 | 83.9% |
|  | Overall | 40 | 7 | 33 | 82.5% |
| TN | SLNB only | 13 | 4 | 9 | 69.2% |
|  | ALND | 35 | 9 | 26 | 74.3% |
|  | Overall | 48 | 13 | 35 | 72.9% |
| Overall | Overall | 324 | 29 | 295 | 91.0% |


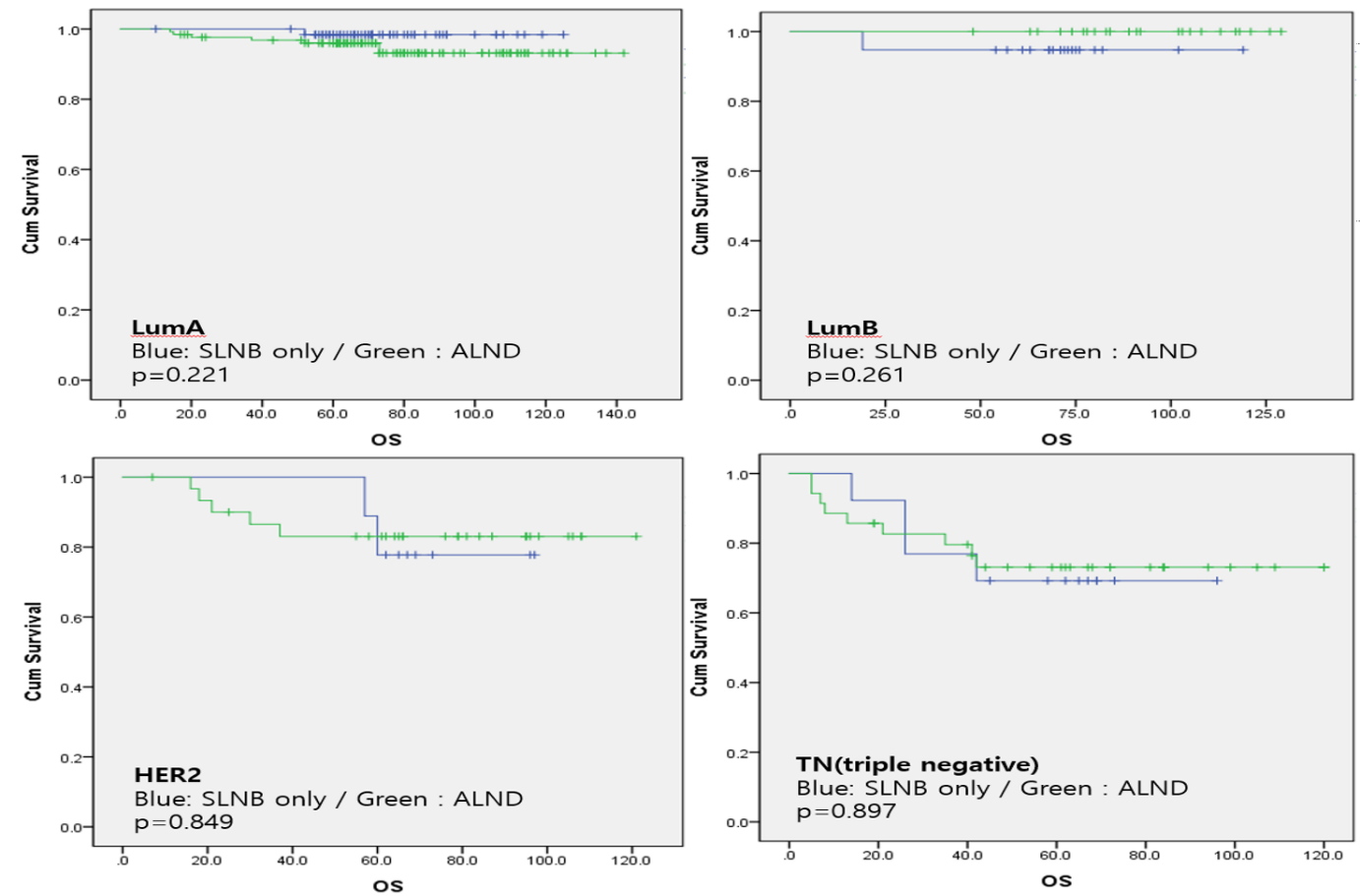

Supplement: Supplementary file 1 — Supplementary Information [file 41598_2021_88442_MOESM1_ESM.docx]
